# MicroRNA-200c coordinates HNF1 homeobox B and apolipoprotein O functions to modulate lipid homeostasis in alcoholic fatty liver disease

**DOI:** 10.1016/j.jbc.2022.101966

**Published:** 2022-04-20

**Authors:** Md Golam Mostofa, Melanie Tran, Shaynian Gilling, Grace Lee, Ondine Fraher, Lei Jin, Hyunju Kang, Young-Ki Park, Ji-Young Lee, Li Wang, Dong-Ju Shin

**Affiliations:** 1 Department of Physiology and Neurobiology, University of Connecticut, Storrs, Connecticut, USA; 2 Department of Nutritional Sciences, University of Connecticut, Storrs, Connecticut, USA; 3 Department of Internal Medicine, Section of Digestive Diseases, Yale University, New Haven, Connecticut, USA

**Keywords:** liver, microRNA, alcohol, lipid, mitochondria, AAV, adeno-associated virus, AFL, alcoholic fatty liver, ALT, alanine transaminase, BW, body weight, CB, LDC without ethanol supplementation plus maltose dextrin binge, CEE, chronic ethanol exposure, EB, LDC supplemented with ethanol plus ethanol binge, FA, fatty acid, LDC, Lieber-DeCarli liquid diet, LC3, light chain 3, MDA, malondialdehyde, miR, microRNA, PC, phosphatidylcholine, PPAR, peroxisome proliferator-activated receptor, TBARS, thiobarbituric acid reactive substances, TEM, transmission electron microscopy, TG, triglyceride, UTR, untranslated region, VLDL, very low density lipoprotein

## Abstract

Hepatic steatosis is an initial manifestation of alcoholic liver disease. An imbalance of hepatic lipid processes including fatty acid uptake, esterification, oxidation, and triglyceride secretion leads to alcoholic fatty liver (AFL). However, the precise molecular mechanisms underlying the pathogenesis of AFL remain elusive. Here, we show that mice deficient in microRNAs (miRs)-141 and -200c display resistance to the development of AFL. We found that miR-200c directly targets HNF1 homeobox B (*Hnf1b*), a transcriptional activator for microsomal triglyceride transfer protein (*Mttp*), as well as apolipoprotein O (*ApoO*), an integral component of the mitochondrial contact site and cristae organizing system complex. We show that expression of these miRs is significantly induced by chronic ethanol exposure, which is accompanied by reduced HNF1B and APOO levels. Furthermore, miR-141/200c deficiency normalizes ethanol-mediated impairment of triglyceride secretion, which can be attributed to the restored levels of HNF1B and MTTP, as well as phosphatidylcholine abundance. Moreover, we demonstrate that miR-141/200c deficiency restores ethanol-mediated inhibition of APOO expression and mitochondrial dysfunction, improving mitochondrial antioxidant defense capacity and fatty acid oxidation. Taken together, these results suggest that miR-200c contributes to the modulation of lipid homeostasis in AFL disease by cooperatively regulating *Hnf1b* and *ApoO* functions.

Alcoholic liver disease is a leading cause of morbidity and mortality, affecting 3.3 million people worldwide annually (1). The initial hepatic manifestation of alcoholic liver disease includes alcoholic fatty liver (AFL), which is characterized by accumulation of fat droplets in hepatocytes (2). Although AFL is reversible, it can progress to more severe liver injury such as hepatitis, fibrosis, cirrhosis, and hepatocellular carcinoma. Despite the considerable progress that has been made to understand the pathophysiology of AFL, the detailed molecular events that underlie the development of the disease have yet to be fully determined.

The pathogenesis of AFL is complex and multifactorial. In liver, lipid homeostasis is maintained by coordinated regulation of fatty acid (FA) uptake, esterification, oxidation, and triglyceride (TG) secretion. Imbalances of these processes can lead to the development of hepatic steatosis. Chronic alcohol consumption increases expression of hepatic cluster of differentiation 36 (CD36) and FA uptake (3), while it up-regulates lipid synthesis by controlling sterol regulatory binding protein-1c (SREBP-1c) and peroxisome proliferator-activated receptor γ (PPARγ) (4, 5). Additionally, chronic alcohol exposure promotes lipid droplet formation by inducing cell death–inducing DFFA like effector c (*Cidec*) and perilipin 2 (*Plin2*) (6, 7). In contrast, reduced FA oxidation and TG secretion have been shown by chronic alcohol consumption. PPARα signaling in FA oxidation is reduced in response to chronic alcohol exposure (8), while hepatic very low density lipoprotein (VLDL) secretion and microsomal triglyceride transfer protein (MTTP) activity are lowered by alcohol consumption (9, 10). Consequently, the disrupted pathways in lipid homeostasis have been linked to the development of AFL, although the precise mechanisms remain elusive.

MicroRNAs (miRs) are small noncoding RNAs that are 22 nucleotides in length, which regulate gene expression at the posttranscriptional level. They bind to target sequences generally located in the 3′ untranslated region (UTR), reducing the stability or translation of their target mRNAs. The miR-200 family comprises five evolutionally conserved members, including miR-200a, miR-200b, miR-429, miR-200c, and miR-141. In mice, miR-200a, miR-200b, and miR-429 are clustered on chromosome 4, while miR-141 and miR-200c are clustered on chromosome 6 (Fig. S1*A*). miR-200b, miR-200c, and miR-429 share the same seed sequences, whereas the seed sequences for miR-200a and miR-141 are the same. There is only one nucleotide that is different in the seed sequences between the two groups, dividing the family members into two functional groups (Fig. S1*B*).

The miR-200 family regulates cell differentiation, apoptosis, epithelial-to-mesenchymal transition, and stem cell maintenance. Consequently, dysregulation of the miR-200 family has been implicated in cancer development and progression (11). Additionally, miR-200b and miR-429 have been shown to be essential for female fertility (12), while the miR-200 family regulates type 2-diabetes–associated pancreatic beta cell survival (13). Moreover, *in vivo* studies have demonstrated the involvement of miR-141 and miR-200c in tooth development (14) and nonalcoholic steatohepatitis (15). However, the functional role of the miR-200 family *in vivo* in AFL remains unknown. Furthermore, the relative functional significance of miR-200 members, especially miR-141 and miR-200c, in regulating AFL has not been uncovered. In this study, we identified that miR-141/200c deficiency prevents AFL. We found that miR-200c directly targets HNF1 homeobox B (*Hnf1b*) and apolipoprotein O (*ApoO*), regulating hepatic TG secretion and mitochondria function. We also provided evidence supporting that miR-200c may be a prominent player in this regulation relative to miR-141.

## Results

### miR-141/200c KO mice display resistance to alcoholic hepatic steatosis

**Figure 1:**
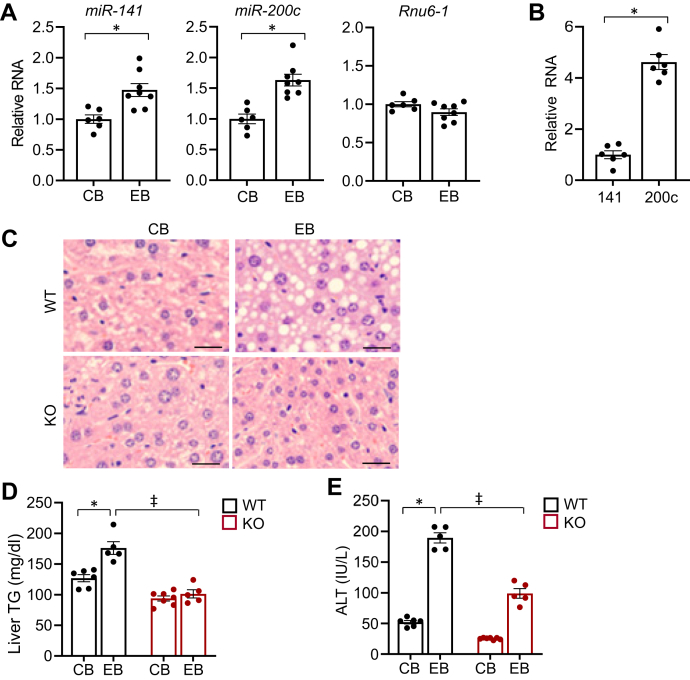
**miR-141/200c KO mice display resistance to alcoholic hepatic steatosis.***A* and *B*, RNA expression levels of miR-141 and miR-200c were measured by qPCR in mouse livers and normalized to *Rnu6-1* (n=6–7). *C*, representative images of H&E staining of liver sections from WT- and KO-fed CB or EB. Images were acquired using a 10× objective. The scale bar represents 100 μm. *D*, hepatic TG content measured by colorimetric assay. *E*, plasma ALT levels. Data are presented as mean ± SEM. Statistical significance was assessed by Student’s *t* test; ∗*p* < 0.05 (*A* and *B*). ∗*p* < 0.05, WT-CB *versus* WT-EB (*D* and *E*); ǂ, *p* < 0.05, WT-EB *versus* KO-EB (*D* and *E*). ALT, alanine transaminase; CB, LDC without ethanol supplementation plus maltose dextrin binge; EB, LDC supplemented with ethanol plus ethanol binge; TG, triglyceride.

To investigate the role of miR-141/200c in alcoholic liver disease, we applied a chronic-plus-binge ethanol-feeding regimen. The single binge of ethanol in combination with chronic ethanol feeding synergistically induces hepatic steatosis. It also causes inflammation and markedly increased plasma alanine transaminase (ALT) levels that peak at 9 h after binge (6, 16, 17). As the feeding regimen induces significant phenotypes of alcoholic liver disease, we used it for the present study to test our hypothesis. Mice were fed a Lieber-DeCarli (LDC) liquid diet supplemented without or with ethanol (5% vol/vol) for 1 month (CB or EB) followed by a single binge of maltose dextrin (9 g/kg body weight, BW) and ethanol (5 g/kg BW), respectively (Fig. S2). Nine hours later, tissues were harvested for RNA and protein analyses. RNA expression of miR-141 and miR-200c was analyzed in livers of the mice. As shown in Figure 1*A*, miR-141 RNA expression was significantly induced by ethanol. Similarly, miR-200c expression levels were higher in mice-fed EB than CB, suggesting a potential role of miR-141 and -200c in the pathogenesis of the disease. RNA, U6 small nuclear 1 (*Rnu6-1*) used as a control showed no significant difference between CB and EB. As miR-141 and miR-200c are closely clustered on the same chromosome, we wondered whether expression levels of miR-141 mirrors those of miR-200c under basal conditions. Interestingly, miR-200c levels were markedly higher than those of miR-141 (Fig. 1*B*). To test the functional significance of miR-141/200c in alcoholic liver disease *in vivo*, we utilized a mouse model where miR-141 and -200c genes were ablated (15). Hepatic steatosis is the hallmark of alcoholic liver disease, and we therefore examined the histology of livers by H&E staining from WT- and KO-mice–fed CB or EB. The histological analysis revealed that ethanol feeding in WT resulted in considerable lipid accumulation in liver, whereas KO-EB mice exhibited fairly normal histology without noticeable lipid droplets (Fig. 1*C*).

To corroborate the histology data, hepatic TG content was analyzed. WT-EB mice displayed increased hepatic TG content compared to WT-CB; however, KO-EB mice maintained the hepatic TG levels similar to those in WT-CB (Fig. 1*D*). Consistent with the hepatic steatosis, WT-EB mice exhibited elevated plasma ALT levels compared to WT-CB mice, indicative of liver damage and injury, while KO-EB mice displayed lower plasma ALT levels than those of WT-EB mice (Fig. 1*E*), demonstrating that miR-141/200c deficiency ameliorates ethanol induced liver injury. Taken together, these results suggest that miR-141/200c KO mice are resistant to develop alcoholic hepatic steatosis and liver injury.

### miR-141/200c deficiency leads to reduced lipid accumulation and altered gene expression in response to ethanol feeding in liver

**Figure 2:**
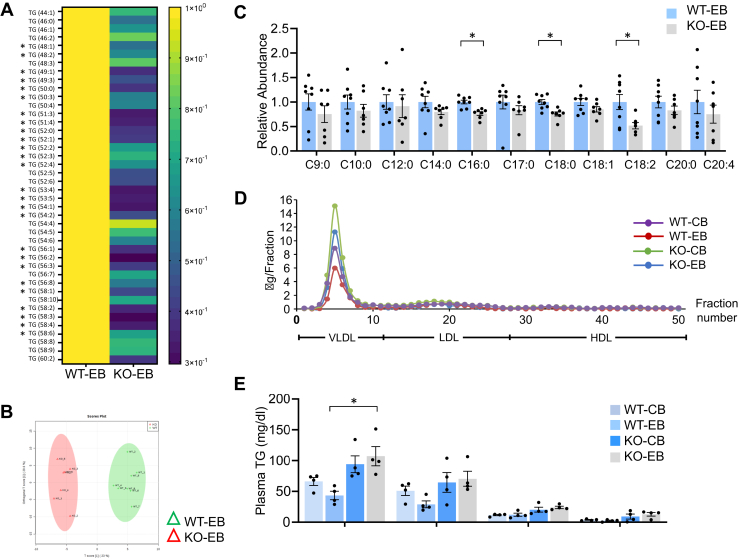
**miR-141/200c deficiency leads to reduced lipid accumulation in response to ethanol feeding in liver.***A*, different TG species were examined in livers of WT-EB and KO-EB by metabolomics and lipidomics analyses (n=7–8). *B*, PLSDA diagram of metabolomics and lipidomics analysis. Liver samples from WT-EB and KO-EB. *C*, different species of fatty acids analyzed by metabolomics in livers of WT-EB and KO-EB. *D*, plasma lipid profiles analyzed by FPLC from WT- and KO-fed CB or EB. *E*, quantification of TGs in plasma lipid profiles in (*D*) is shown. Data are presented as mean ± SEM. Statistical significance was assessed by Student’s *t* test; ∗*p* < 0.05. PLSDA, Partial least squares discriminant analysis; FPLC, fast protein liquid chromatography; CB, LDC without ethanol supplementation plus maltose dextrin binge; EB, LDC supplemented with ethanol plus ethanol binge; TG, triglyceride.

We further investigated hepatic lipid profiles by metabolomics and lipidomics analyses. The analyses revealed that many different species of hepatic TGs were significantly reduced in KO-EB, compared to WT-EB (Fig. 2*A* and Table S1). Partial least squares discriminant analysis providing a visualization of the data on a 2-dimensional map demonstrated that the data of the two groups were well separated and differentiated, verifying the analysis (Fig. 2*B*). In addition, abundances of hepatic long-chain fatty acids (LCFAs), including palmitic acid (C16:0), stearic acid (C18:0), and linoleic acid (C18:2), were significantly lower in KO-EB than WT-EB, while the abundance of medium-chain FAs which are less obesogenic than LCFAs was comparable between WT-EB and KO-EB (Fig. 2*C*).

To further characterize the phenotype of miR-141/200c deficiency in the context of alcoholic liver steatosis, TG content in plasma was analyzed by fast protein liquid chromatography. WT-EB showed a reduced trend in total plasma TG content compared to WT-CB (*p* = 0.052), which was restored by miR-141/200c deficiency. Plasma TG content in VLDL fractions tended to be lower in WT-EB than WT-CB but did not reach statistical significance (Fig. 2*E*).

**Figure 3:**
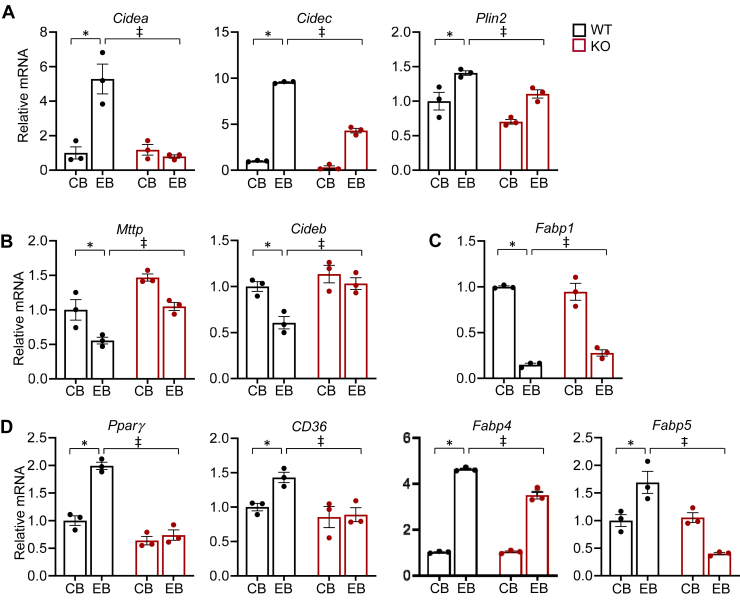
**miR-141/200c deficiency leads to altered expression of genes in hepatic lipid metabolism in response to ethanol feeding**. *A*–*D*, mRNA expression levels of genes were measured by qPCR in mouse livers and normalized to *Rpl32* or *Hprt* (n=6–8). Data are presented as mean ± SEM. Statistical significance was assessed by Student’s *t* test; ∗*p* < 0.05, WT-CB *versus* WT-EB; ǂ, *p* < 0.05, WT-EB *versus* KO-EB. CB, LDC without ethanol supplementation plus maltose dextrin binge; EB, LDC supplemented with ethanol plus ethanol binge.

To investigate whether the changes in hepatic and plasma lipid profiles are linked to alterations in gene expression, we analyzed expression of genes in lipid metabolism. Expression of *Cidea* and *Cidec* involved in lipid droplet formation was markedly increased in WT-EB compared to WT-CB, which was significantly inhibited by miR-141/200c deficiency. The expression pattern of *Plin2*, a scaffold protein of lipid droplets, was similar to that of *Cidea* and *Cidec* (Fig. 3*A*).

In contrast to the increased expression of genes in Figure 3*A*, we observed significantly reduced expression of *Mttp*, a lipid transfer protein required for the assembly and secretion of VLDL, by ethanol feeding in WT mice compared to WT-CB, whereas miR-141/200c deficiency restored the reduced gene expression to that of WT-CB (Fig. 3*B*). Similarly, expression of *Cideb*, a member of the CIDE protein family in VLDL lipidation and maturation, was down-regulated by ethanol in WT; however, KO-EB mice displayed normal levels of *Cideb* similar to those of WT-CB, which were significantly higher than those of WT-EB (Fig. 3*B*). Moreover, mRNA levels of FA binding protein 1 (*Fabp1*), an intracellular FA binding protein, were markedly reduced by EB compared to CB in WT, while miR-141/200c deficiency partially restored the ethanol-mediated inhibition of *Fabp1* mRNA expression (Fig. 3*C*)

Additionally, expression of *Pparγ*, *Cd36*, *Fabp4*, and *Fabp5*, which are involved in FA synthesis, uptake, and transport, was highly upregulated by ethanol feeding in WT compared to WT-CB; however, KO-EB mice displayed partial or complete inhibition of the ethanol-mediated induction of gene expression (Fig. 3*D*). Overall, the alterations of gene expression in lipid metabolism are in agreement with the results from the liver histology and the hepatic and plasma lipid profiles.

### miR-200c directly targets *Hnf1b* and *ApoO*

**Figure 4:**
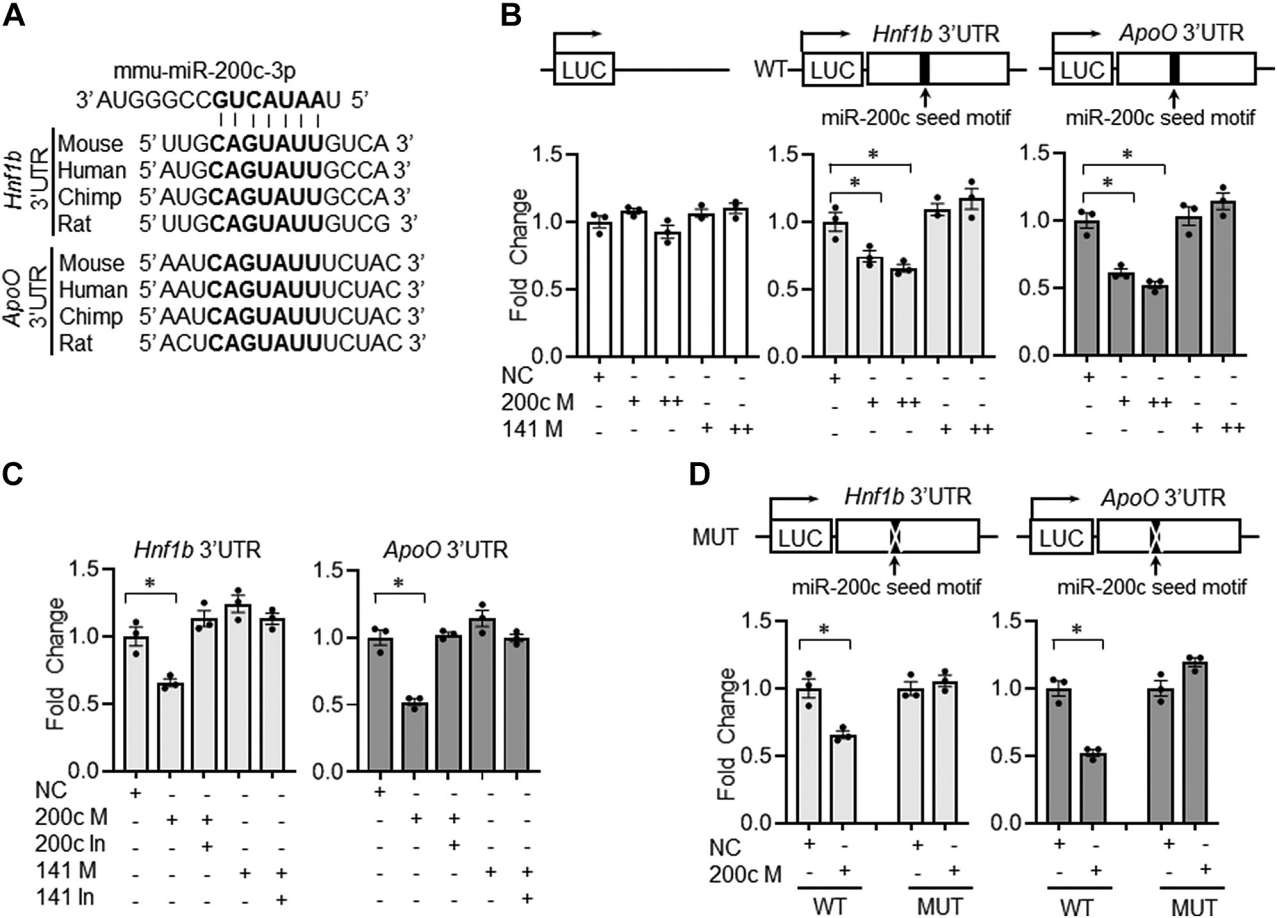
**miR-200c directly targets *Hnf1b* and *ApoO in vitro*.***A*, alignment of putative miR-200c seed sequences in *Hnf1b* and *ApoO* 3′ UTRs. The reference sequence of mouse miR-200c-3p is shown. The seed sequence of miR-200c is shown in *bold*. *B* and *C*, luciferase reporter assays of control luciferase reporter (pmirGLO), *Hnf1b*-3′UTR-Luc, and *ApoO*-3′UTR-Luc in Hepa1 cells. Ten nanomolar or twenty-five nanomolar of microRNA mimic or inhibitor was used for transfection assays. *D*, luciferase reporter assays of mutant *Hnf1b*-3′UTR-Luc and *ApoO*-3′UTR-Luc in Hepa1 cells. Twenty-five nanomolar of microRNA mimic was used for transfection assays. Statistical significance was assessed by Student’s *t* test; ∗*p* < 0.05. Luc, luciferase; NC, nonspecific control; M, mimic; In, inhibitor. UTR, untranslated region.

Given the inverse relationship between the hepatic and plasma TG levels with the accompanying gene expression data, we considered the possibility that miR-141/200c could regulate genes in TG secretion, participating in hepatic steatosis. MTTP plays key role in TG secretion, but considering that MTTP activity is primarily regulated at the transcriptional level (18), we hypothesized that miR-141/200c regulates *Mttp* expression indirectly. Since HNF1A, HNF1B, and hepatocyte nuclear factor 4 (HNF4) are critical transcription factors for *Mttp* transcription (18), we examined 3′UTR sequences of the genes using the TargetScan search program (19). We found that *Hnf1b* contains a highly conserved putative miR-200c seed sequence, but not a miR-141 seed sequence (Fig. 4*A* top). To validate the putative miR-200c seed sequence, we performed luciferase reporter assays with the construct containing the *Hnf1b* 3′UTR. Reporter assays revealed that the *Hnf1b* 3′UTR activity was reduced by miR-200c mimic in a dose-dependent manner, which was reversed by miR-200c inhibitor (Fig. 4, *B* middle and *C* left). In contrast, the mutant *Hnf1b* 3′UTR failed to respond to miR-200c mimic (Fig. 4*D* left). The *Hnf1b* 3′UTR activity was responsive to neither miR-141 mimic nor inhibitor (Fig. 4, *B* middle and *C* left). Luciferase activity of the control vector without the seed sequence was not changed by miR-141 or miR-200c mimic (Fig. 4*B* left).

Apolipoproteins are structural components of lipoproteins and are involved in the transport of TGs from the liver. To explore the role of miR-141/200c in regulating apolipoproteins, 3′UTRs of hepatic apolipoproteins present in VLDL, including *Apolipoprotein (Apo) B*, *ApoC1*, *ApoC2*, *ApoC3*, *ApoE*, and *ApoO*, were examined. The analysis revealed that none of the genes tested except *ApoO* harbor a highly conserved putative miR-200c seed sequence (Fig. 4*A* bottom). A putative miR-141 seed sequence was not found in any of the genes tested. To test whether the miR-200c seed sequence is functional, similar experiments were performed using a luciferase reporter containing the *ApoO* 3′UTR. miR-200c mimic significantly lowered the *ApoO* 3′UTR activity, which was reversed by miR-200c inhibitor (Fig. 4, *B* right and *C* right). The inhibitory effect of miR-200c was abrogated by the introduction of mutation to the seed sequence (Fig. 4*D* right). There was no effect of miR-141 mimic or inhibitor on the *ApoO* 3′UTR activity (Fig. 4*B* right and *C* right).

**Figure 5:**
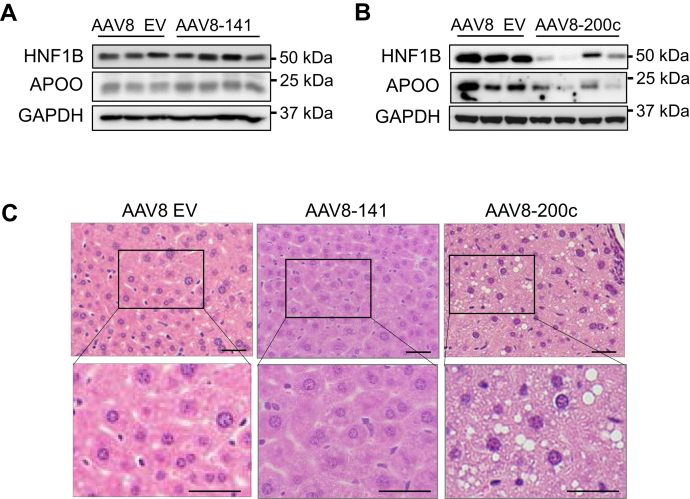
**miR-200c targets *Hnf1b* and *ApoOin vivo***. *A* and *B*, Western blot of protein prepared from mouse livers overexpressing miR-141 (AAV8-141, A) or miR-200c (AAV8-200c, B). *C*, representative images of H&E staining of liver sections from mice overexpressing miR-141 or miR-200c. Images were acquired using a 10× objective. The scale bar represents 100 μm. EV, empty vector; AAV, adeno-associated virus; miR, microRNA.

To follow up the *in vitro* data, we overexpressed either miR-141 or miR-200c primarily in livers of WT mice by intravenous delivery of adeno-associated virus (AAV) 8 with the liver specific TBG promoter. Overexpression of miR-141 and miR-200c was verified (Fig. S3). Immunoblot analyses revealed that while miR-141 overexpression resulted in comparable expression of hepatic HNF1B and APOO between the two groups (Fig. 5*A*), miR-200c overexpression significantly reduced the hepatic expression of HNF1B and APOO (Fig. 5*B*). Additionally, H&E staining demonstrated that mice overexpressed with miR-141 displayed normal liver histology, whereas miR-200c overexpression resulted in noticeable lipid deposition (Fig. 5*C*). Overall, these data suggest that miR-200c directly regulates hepatic *Hnf1b* and *ApoO*, contributing to hepatic fat accumulation and that miR-141 may not be involved in this regulation.

### miR-141/200c deficiency normalizes ethanol-mediated hepatic TG secretion

**Figure 6:**
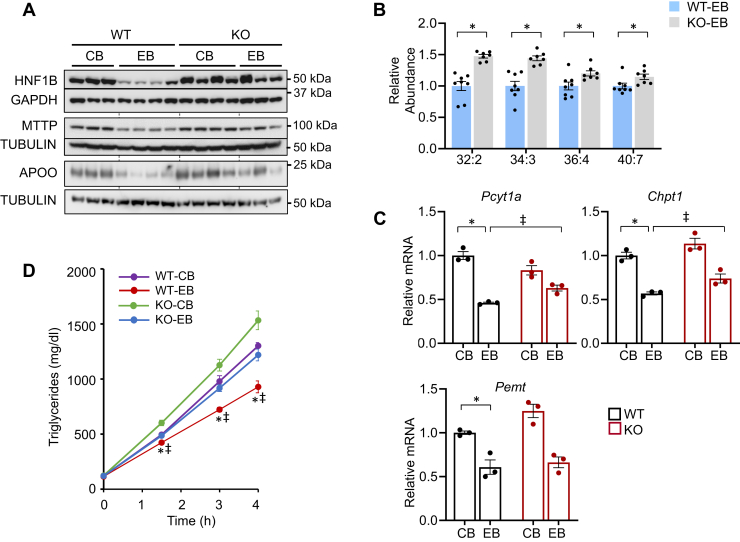
**miR-141/200c deficiency normalizes ethanol-mediated reduction of hepatic TG secretion and PC abundance.***A*, Western blot of protein prepared from WT- and KO-mice–fed CB or EB. *B*, different species of PCs measured by lipidomics analysis. *C*, mRNA expression levels of genes were measured by qPCR in mouse livers and normalized to *Rpl32* or *Hprt* (n=6–8). *D*, TG secretion rates were analyzed over a 4 h period after intravenous injection of Triton WR-1339 (500 mg/kg BW) in WT- and KO-fed CB or EB (n=4–8). Data are presented as mean ± SEM. Statistical significance was assessed by Student’s *t* test; ∗*p* < 0.05 (*B*). ∗*p* < 0.05, WT-CB *versus* WT-EB (*C* and *D*); ǂ, *p* < 0.05, WT-EB *versus* KO-EB (*C* and *D*). BW, body weight; CB, LDC without ethanol supplementation plus maltose dextrin binge; EB, LDC supplemented with ethanol plus ethanol binge; PC, phosphatidylcholine; TG, triglyceride.

To further investigate the role of miR-141/200c *in vivo* in regulating TG secretion in alcoholic liver disease, we examined protein levels of HNF1B, MTTP, and APOO in WT- and KO-mice–fed CB or EB. Immunoblot analyses showed that HNF1B protein levels and its downstream target, MTTP, were lower in WT-EB than WT-CB, whereas miR-141/200c deficiency resulted in normalizing the ethanol-mediated reduction of the protein to those of control mice. Similarly, APOO protein levels were significantly reduced by ethanol feeding in WT-EB compared to WT-CB; however, KO-EB mice displayed restored APOO, which was otherwise reduced by chronic ethanol exposure (CEE) (Fig. 6*A*).

The VLDL surface monolayer surrounding the core lipids consists of phospholipids and cholesterol along with apolipoproteins. Phosphatidylcholine (PC) is the most abundant phospholipid, comprising 60 to 80% of total phospholipids. Consistently, hepatic VLDL secretion is impaired by reduced PC abundance (20). Therefore, hepatic PC levels were examined by lipidomics analysis in WT- and KO-mice–fed EB. The abundance of several species of PCs (32:2, 34:3, 36:4, and 40:7) were significantly higher in KO-EB than WT-EB (Fig. 6*B*). PCs are synthesized by two pathways mainly in liver (21). The major pathway is the CDP-choline pathway where phosphate cytidylyltransferase 1A, choline (PCYT1a) serves as the rate-limiting enzyme. The second pathway involves phosphatidylethanolamine N-methyltransferase (PEMT), which produces PCs endogenously from phosphatidylethanolamine. Gene expression analysis revealed that ethanol feeding significantly reduced *Pcyt1a* mRNA levels in WT-EB compared to WT-CB, whereas miR-141/200c deficiency restored the ethanol-mediated reduction of *Pcyt1a* expression (Fig. 6*C*). Similar expression patterns were observed for choline phosphotransferase 1 (*Chpt1*), which is involved in the CDP-choline pathway. Additionally, expression of *Pemt* was lower in WT-EB than WT-CB; however, *Pemt* mRNA levels were similar between WT-EB and KO-EB.

To directly verify the role of miR-141/200c *in vivo* in hepatic TG secretion, mice were administrated intravenously with Triton WR-1339, an inhibitor of lipoprotein lipase, and TG secretion rates were analyzed over a 4 h period. Under these conditions, TGs accumulate in serum at a rate as they are produced due to the inhibition of TG hydrolysis. We observed an increase in TGs in serum over time following Triton WR-1339 administration (Fig. 6*D*). However, the TG secretion rate was lower in WT-EB than WT-CB, while miR-141/200c deficiency normalized the ethanol-mediated reduction of TG secretion to that observed in WT-CB.

### miR141/200c deficiency improves ethanol-induced mitochondrial dysfunction

**Figure 7:**
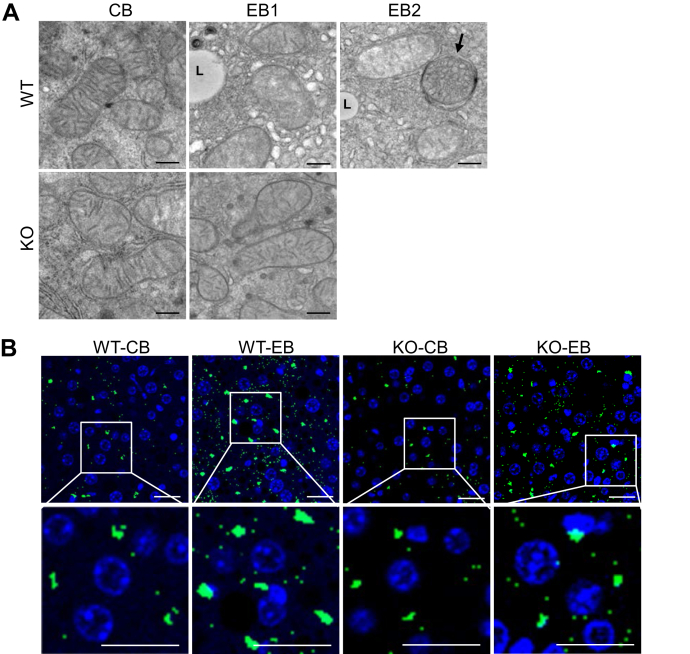

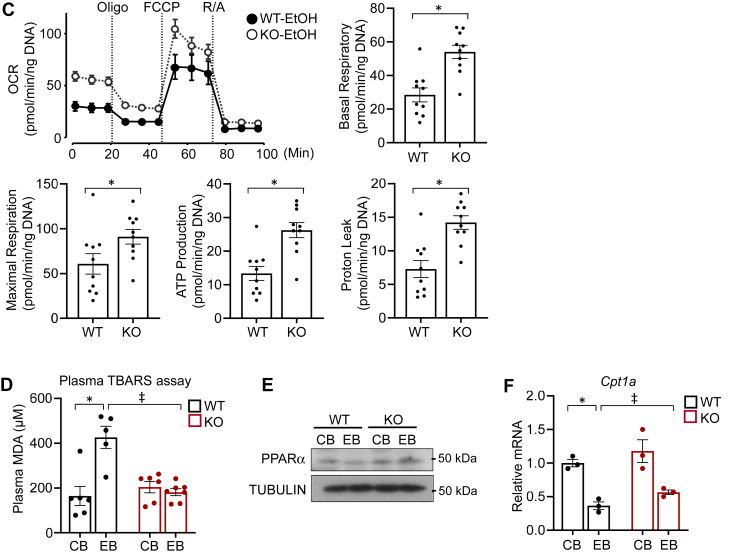
**miR141/200c deficiency improves ethanol-induced mitochondrial dysfunction.***A*, representative images of TEM of liver sections from WT- and KO-fed CB or EB. Images were acquired at x23,000 magnification. The scale bar represents 500 nm. *B*, representative images of LC3 immunofluorescence of liver sections from WT- and KO-fed CB or EB. Images were acquired at the 40× oil immersion objective. The scale bar represents 50 μm. *C*, OCR of primary hepatocytes prepared from WT- and KO-fed LDC supplemented with ethanol for 1 month was analyzed by Seahorse FX24 analyzer under basal conditions or in response to indicated inhibitors. *D*, a plasma TBARS assay is shown. *E*, Western blot of protein pooled from WT- and KO-fed CB or EB (n=6–8). *F*, mRNA expression levels of genes were measured by qPCR in mouse livers and normalized to *Rpl32* or *Hprt* (n=6–8). Data are presented as mean ± SEM. Statistical significance was assessed by Student’s *t* test; ∗*p* < 0.05 (*C*). ∗*p* < 0.05 WT-CB *versus* WT-EB (*D* and *F*); ǂ, *p* < 0.05, WT-EB *versus* KO-EB (*D* and *F*). The *arrow* in (*A*) indicates an autophagosome. L, lipid droplet; OCR, oxygen consumption rate; Oligo, oligomycin; FCCP, Carbonyl cyanide-4 (trifluoromethoxy) phenylhydrazone; R/A, rotenone and antimycin A. CB, LDC without ethanol supplementation plus maltose dextrin binge; EB, LDC supplemented with ethanol plus ethanol binge; LDC, Lieber-DeCarli liquid diet; OCR, oxygen consumption rate; TBARS, thiobarbituric acid reactive substances; TEM, transmission electron microscopy; LC3, microtubule-associated protein 1 light chain 3.

Although *ApoO* is a member of the apolipoprotein family, interestingly, it is localized in the mitochondria where it serves as an integral component of the mitochondrial contact site and cristae organizing system complex (22). Since chronic alcohol exposure is associated with mitochondrial dysfunction (23), we investigated the effects of miR-141/200c deficiency on mitochondrial function in the context of alcoholic liver disease. As the mitochondrial contact site and cristae organizing system is responsible for maintaining mitochondrial architecture and crista junctional integrity (22), we examined ultrastructure of mitochondria in livers of mice-fed CB or EB by transmission electron microscopy (TEM). Compared to WT-CB, ethanol feeding in WT showed signs of structural damage to mitochondria. WT-EB mice displayed reduced crista abundance and twirled arrangement (Figs. 7*A* and S4, WT-EB1), in contrast to normal lamellar cristae shown in WT-CB. Furthermore, around swollen mitochondria that contain less electron dense matrices, we observed autophagosomes which appear when increased accumulations of damaged mitochondria are present (Figs. 7*A* and S4, WT-EB2). In contrast, KO-EB mice displayed similar mitochondrial morphology and ultrastructure to those of WT-CB. The formation and maturation of autophagosomes require microtubule-associated protein 1 light chain 3 (LC3), which plays a key role in autophagy, a catabolic degradative process of removing unnecessary dysfunctional cellular components. Therefore, LC3 serves as a specific marker of autophagosomes (24). Confocal microscopy of LC3 immunofluorescence revealed that WT-EB mice exhibit increased LC3 staining compared to WT-CB, while miR-141/200c deficiency resulted in diminished LC3 staining (Fig. 7*B*), suggesting that livers of WT-EB display increased autophagic activity, which would be associated with an increase in damaged mitochondria.

To directly analyze the function of mitochondria, primary hepatocytes were prepared from WT-mice–fed LDC supplemented without (WT-Ctrl) or with ethanol (WT-EtOH) for 1 month, and mitochondria functional assays were performed by Seahorse XF24 Analyzer. We analyzed oxygen consumption rate representing mitochondrial respiration activity and found that WT-EtOH hepatocytes displayed lower basal respiration than WT-Ctrl cells (Fig. S5). Similarly, there was a significant reduction of maximal respiration in WT-EtOH hepatocytes compared to those of WT-Ctrl, when hepatocytes were treated with carbonyl cyanide-4 (trifluoromethoxy) phenylhydrazone, an uncoupling agent that induces maximal respiration. Additionally, proton leak and ATP production which represents the capacity of mitochondria to meet cell’s energy needs were significantly lower in WT-EtOH hepatocytes than those of WT-Ctrl. These data suggest that ethanol treatment reduced mitochondrial respiration and capacity. To evaluate the effects of miR-141/200c deficiency on mitochondrial function under CEE, similar experiments were carried out in hepatocytes isolated from WT (WT-EtOH)- and KO (KO-EtOH)-mice–fed LDC supplemented with ethanol for 1 month. As shown in Figure 7*C*, miR-141/200c deficiency increased basal and maximal respiration compared to WT-EtOH. Additionally, KO-EtOH hepatocytes exhibited higher proton leak and ATP production compared to WT-EtOH hepatocytes.

Mitochondria play critical roles in generating cellular energy and are involved in intermediary metabolism and oxidative stress defense. Impaired mitochondrial function is associated with increased oxidative stress (23). To analyze whether damaged mitochondria are associated with alterations in antioxidant defense capacity, we performed the thiobarbituric acid reactive substances (TBARS) assay, which detects malondialdehyde (MDA), the major lipid oxidation product. A plasma TBARS assay revealed that WT-EB mice exhibited higher plasma MDA levels than those of WT-CB mice, which was fully restored by miR-141/200c deficiency (Fig. 7*D*). Moreover, protein levels of PPARα, an important transcription factor involved in mitochondria metabolic functions including FA oxidation (25), were reduced by CEE in WT mice, while KO-EB mice displayed reversed PPARα levels compared to WT-EB (Fig. 7*E*). Consistently, mRNA expression of carnitine palmitoyltransferase 1 a (*Cpt-1a*), a PPARα target gene in FA oxidation (26), was lower in WT-EB than WT-CB, which was partially reversed by miR-141/200c deficiency (Fig. 7*F*). Overall, these data suggest that CEE induces mitochondrial dysfunction and oxidative stress, and reduces FA oxidation, whereas miR-141/200c deficiency contributes to restoring the ethanol-induced mitochondrial damage and function.

## Discussion

**Figure 8:**
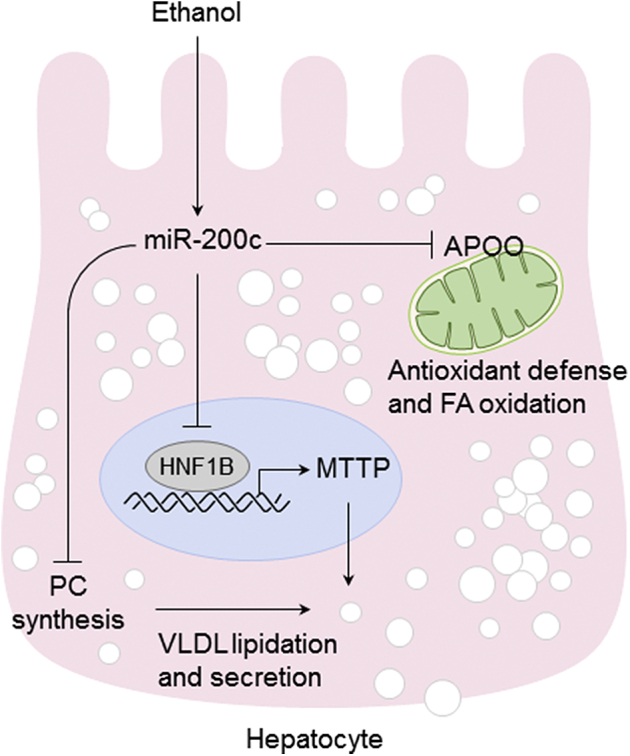
**Model for miR-200c–mediated regulatory pathways in alcoholic hepatic steatosis**.

In the present study, we identified novel targets of miR-200c, *Hnf1b*, *and ApoO*, regulating TG secretion and mitochondrial function, providing previously unappreciated mechanisms for alcoholic hepatic steatosis (Fig. 8). Earlier studies have shown that ethanol reduces MTTP activity accompanied by increased hepatic lipid content (10, 27); however, the underlying mechanisms remained elusive. *Mttp* mRNA levels reflect the MTTP protein and activity levels (18). The proximal promoter region of *Mttp* is highly conserved and harbors multiple regulatory elements for transcription factors such as HNF1 and HNF4. As the transcription of *Mttp* is under the control of various hormones and nutrients, ethanol reduces the levels of mRNA, protein, and activity of MTTP. The down-regulation of MTTP activity appeared to occur at the transcriptional level (27). Consistently, our study suggests that miR-200c regulates *Mttp* transcription indirectly through the direct targeting of *Hnf1b* in response to ethanol (Figs 3*B* and 4, and 6*A*).

It was initially identified that mutations in *HNF1B* are linked to the development of maturity-onset diabetes of the young types 5 (28), while variants of *HNF1B* contribute to susceptibility to type 2 diabetes (29). A growing body of evidence suggests the involvement of HNF1B in lipid homeostasis. *Hnf1b* knockdown reduces insulin sensitivity and glucose tolerance (30) and increases hepatic lipid accumulation (31). In contrast, HNF1B overexpression alleviates lipid accumulation in high-fat–induced diabetic mice and leptin receptor-deficient *db/db* mice. HNF1B protein levels were lower in livers of diet-induced obese mice and *db/db* mice. Our findings are in agreement with previous studies in regard to the reduced HNF1B associated with hepatic steatosis. Genetic loss of *Hnf-4* in liver abolishes MTTP expression in mice, leading to the accumulation of lipids in the liver (32), indicating that HNF-4 is a critical transcription factor for *Mttp*. However, in our experimental setting, HNF-4 may not be involved in regulating *Mttp*, as our sequence screening searching for putative miR-141 or miR-200c binding sites was unsuccessful. Nevertheless, the increased basal levels of *Mttp* in KO-CB (Fig. 3*B*) suggest that miR-141/200c may target other transcription factors that regulate basal transcription of *Mttp*.

FABPs facilitate intracellular FA transport and storage. They bind to unesterified long-chain fatty acids and other ligands (33). FABP1 is highly abundant in the liver. Smathers et al. have found that *Fabp1* KO mice are susceptible to oxidative stress in early-stage alcoholic liver and that FABP1 levels were inversely correlated with the severity of fatty liver (34). In accordance, we observed markedly lower *Fabp1* mRNA expression in EB than CB in WT, which was partially restored in KO-EB (Fig. 3*C*). Interestingly, *Fabp1* is induced by high-fat liquid diet overfeeding through catheter infusion compared to high-fat diet feeding (35). Additionally, *Fabp1* is increased in simple steatosis (128%), when compared to obese normal patients; however, it is under-expressed in nonalcoholic steatohepatitis (36), suggesting that FABP1 may function in a context-dependent manner. *Fabp4* mRNA expression has been shown highly induced by chronic alcohol exposure (37), consistent with our observations (Fig. 3*D*).

It has been shown that patients with alcoholic liver disease display higher free FA levels than patients with morbid obesity (38). Moreover, ethanol treatment inhibits FA oxidation, leading to an increased availability of long-chain fatty acids for the esterification of TGs in hepatocytes (39). Palmitic acid (C16:0) and stearic acid (C18:0) are the most abundant and common long chain fatty acids in the body (40). In line with previous findings, deficiency of miR-141/200c was associated with lower levels of palmitic and stearic acids in response to ethanol than WT-EB (Fig. 2*C*). Interestingly, linoleic acid (C18:2) levels also appeared to be lower in KO-EB than WT-EB. Linoleic acid has been shown to be beneficial to cardiovascular health; however, recent studies have raised concerns about detrimental effects of elevated dietary linoleic acid on inflammation and cancer (41). Further studies to identify the functional role of individual FAs in lipid metabolism are warranted.

Expression of miR-141 and miR-200c is induced in livers of nonalcoholic fatty liver disease (42) and steatohepatitis (15) and in islets of diabetic mice (13). Additionally, overexpression of miR-141/200c induces differentiation of ST2 cells, bone marrow-derived stroma cells, into lipid-loaded adipocytes (43). These studies suggest the important role of miR-141/200c in regulating metabolic pathways. miR-141 and miR-200c are intergenic miRs and their transcription is regulated in a similar fashion by their own promoter regulatory unit, which is well conserved with the presence of core regulatory elements (15, 44). In accordance, we observed increased transcription of miR-141 and miR-200c by ethanol (Fig. 1*A*). Interestingly, we found markedly higher miR-200c RNA levels than those of miR-141 under normal conditions, demonstrated by TaqMan qPCR assay with multiplexed reverse transcription (Fig. 1*B*). miR-200c is separated from miR-141 by a 336 base-pair spacer. The spacer contains two putative response elements for zinc-finger E-box binding homeobox 1 (ZEB1) (45), a well-established transcriptional repressor regulating miR-141 and miR-200c, which raises the possibility that the ZEB1-binding elements could participate in the repression of miR-141. Indeed, miR-141 expression levels were significantly lower than miR-200c in ameloblasts (14). Whether this differential expression pattern of miR-141 and miR-200c is universal in other tissues remains unclear. Although both miR-141 and miR-200c are in the same family, they have a different seed sequence, implying a possibility that they have their own unique targets and function. Some genes harbor seed sequences for both microRNAs and others have either one. Future studies may shed new light on the respective expression and function of miR-141 and miR-200c.

Studies have shown that knockdown of *ApoO* alters mitochondrial architecture and impairs mitochondrial function (46). In accordance, we found dysfunctional mitochondria when APOO levels were reduced by ethanol. Initial studies have shown that APOO was found in serum in association with high density lipoprotein and to a lesser extent with low density lipoprotein, and VLDL (47). In diabetic human heart, *APOO* expression is induced and the expression levels are positively correlated with those of *MTTP*. Moreover, APOO secretion requires MTTP activity in *in vitro* studies. Based on the findings, Lamant et al. proposed that APOO functions as a secreted protein involved in lipoprotein metabolism. Therefore, the initial hypothesis of our study was that APOO plays a role in regulating TG secretion with MTTP. However, transgenic mice overexpressing APOO exhibit no significant alterations in high density lipoprotein and cholesterol metabolism (48). Furthermore, the APOO found in our study is a nonglycosylated form with a molecular mass of 22 kDa, which is smaller than glycosylated APOO (55 kDa) present in lipoproteins. Nonglycosylated APOO is found in mitochondria. Moreover, the nonglycosylated APOO appeared as the major form in our study. Therefore, we propose that APOO is involved in regulating mitochondrial function and FA oxidation. A recent study has shown that overexpression of *ApoO* by recombinant adenovirus in mice results in dysfunctional mitochondria and lipid accumulation (49). Since both overexpression and knockdown of *ApoO* impair mitochondrial function (46), future studies will require to address in more detail how APOO regulates mitochondrial function and contributes to fatty liver disease under certain pathophysiological conditions.

The CDP-choline pathway accounts for 70% of PC biosynthesis in the liver (21). The importance of the CDP-choline pathway in PC biosynthesis and VLDL production has been demonstrated by a study using a liver-specific *Pcyt1a* KO mouse model, displaying increased hepatic TG accumulation, and reduced PC mass, VLDL secretion, and plasma TG. Notably, *Pcyt1a* KO mice showed enhanced PEMT expression, which was insufficient to restore the reduced hepatic VLDL secretion. Therefore, *Pcyt1a* appears to be required for normal secretion of VLDL (50). The PEMT pathway accounts for 30% of PC biosynthesis in liver. It has been shown that CEE reduces PEMT activity, leading to reduced hepatic PC generation and VLDL secretion (51). Accordingly, male *Pemt* KO mice exhibited hepatic lipid accumulation and reduced VLDL secretion and plasma TG levels (52). Consistent with the critical role of *Pcyt1a* in PC biosynthesis, our study suggests that miR-141/200c may function in the CDP-choline pathway rather than the PEMT pathway, regulating PC biosynthesis and TG secretion.

The impaired TG secretion in WT-EB in our study (Fig. 6*D*) was accompanied by lower plasma TG levels. This has been previously described in studies with rats and humans treated with ethanol (53, 54). In contrast, other studies have shown increased TG secretion and hypertriglyceridemia by ethanol treatment (55, 56). There are many factors that can influence phenotypic characteristics of alcoholic liver disease such as drinking patterns and frequency, alcohol doses, administration routes, nutritional status, genetics, and age. In an isolated liver perfusion study, high concentrations of ethanol (4 mg/ml) resulted in inhibition of hepatic TG release (57), whereas lower concentrations of ethanol (2 mg/ml) displayed unaltered hepatic TG release (58). Additionally, in a human study, an initial elevation of serum TG levels was demonstrated for 2 weeks in a 25-days prolonged ethanol administration. However, when blood ethanol concentrations were further increased as the ethanol administration continued, serum TG levels were reduced to or below the initial levels. The implications of the studies suggest that serum TG concentrations could be inversely related to blood alcohol concentrations. Alcoholic hypertriglyceridemia is considered a transient and recurrent condition following excessive drinking (59). Therefore, we propose that in our experimental setting, the binge ethanol administration could contribute to reducing TG secretion, exaggerating the existing hepatic steatosis that has been developed by chronic ethanol administration. Indeed, markedly increased fat accumulation occurred in mouse livers by 8 weeks of ethanol feeding with one binge, compared to those without binge administration (6). Further studies are warranted to better understand the underlying mechanism.

In conclusion, the present study demonstrates a novel function of miR-200c directly targeting *Hnf1b* and *ApoO* to regulate hepatic TG secretion and mitochondrial function modulating lipid homeostasis in AFL. In view of the small seed region, microRNAs target hundreds of genes and the possibility remains that miR-141/200c target other genes involved in lipid metabolism. More research will be needed to better understand the fine-tuning of intricate metabolic networks governed by miR-141/200c.

## Experimental procedures

### Animals

miR-141/200c KO mice were described previously (15). All mice were housed in a light- and temperature-controlled facility and fed *ad libitum* with a standard diet unless otherwise specified. For ethanol feeding, eight- to 10-week-old male mice were fed an LDC diet supplemented with ethanol (5% vol/vol) for 1 month followed by a single binge of ethanol (5 g/kg body weight (BW), EB) (16). For isocaloric control pair feeding, mice were fed an LDC without alcohol for 1 month, followed by a single binge of maltose dextrin (9 g/kg BW, CB). Both groups of mice were fed the LDC for 5 days to acclimatize to liquid-tube feeding prior to alcohol feeding. At 9 h postbinge, mice were sacrificed, and tissues and blood were collected for analysis of RNA, protein, and other metabolic parameters (Fig. S2). For overexpression of miR-141 or miR-200c in liver, mice were administrated with AAV-8-mmu-mir-141 or AAV8-mmu-mir-200c at a dose of 1 × 10^11^ genome copies/mouse by intravenous injection. Four to 6 weeks later, livers were harvested for RNA and protein analysis. All animal procedures were approved and performed in accordance with the guidelines of the Institutional Animal Care and Use Committee of the University of Connecticut.

### Cell culture and transfection assay

Hepa1 cells were maintained in Dulbecco's modified Eagle's medium supplemented with 10% fetal bovine serum at 37 °C and 5% CO_2_. Transient transfection was performed using X-tremeGENE HP DNA Transfection Reagent (Sigma), according to the manufacturer’s instructions. Briefly, cells were seeded in 24-well dishes 1 day before transfection. Twenty-four hours later, cells were transfected with 200 ng/well of the indicated luciferase-reporter, 200 ng/well of renilla luciferase reporter (pRL-TK, Promega), and microRNA mimic or inhibitor (ThermoFisher Scientific) at a final concentration of 10 nM or 25 nM. Twenty-four hours later, cells were harvested and luciferase activity was analyzed using the Dural-Luciferase Reporter Assay System (Promega) where the firefly luciferase activity was normalized to renilla luciferase activity to control the variation of transfection efficiency and cell growth. Values represent the mean of triplicates ± SEM.

### Plasmids

A fragment (294 bp) of mouse *ApoO* 3′UTR containing a putative miR-200c–binding site was inserted into the NheI and SalI sites downstream of the luciferase in the pmirGLO (Promega). To construct an *Hnf1b* 3′UTR reporter, a fragment (395 bp) of mouse *Hnf1b* 3′UTR containing a putative miR-200c–binding site was inserted into the NheI and SalI sites downstream of the luciferase in the pmirGLO (Promega). Mutations were introduced to the putative miR-200c–binding sites by Site-Directed Mutagenesis (Agilent Technologies) to generate mutant constructs (Fig. S6). The nucleotide sequences of the constructs were verified by DNA sequencing analysis.

### Histological analysis

Liver was fixed in 10% buffered formalin, embedded in paraffin, sectioned at 4 μm, and stained with H&E. Images were obtained by an Olympus BX43 Light Microscope using the Olympus cellSens imaging software.

### Plasma TG, ALT, and TBARS measurements

Plasma TG levels were analyzed colorimetrically using the Triglyceride assay kit (Pointe Scientific). Plasma ALT levels were analyzed by the Infinity ALT assay kit, according to the manufacturer’s instructions. Plasma TBARS assays were performed by measuring MDA levels as described previously (60)

### Liver TG

Liver TGs were extracted by the chloroform-methanol Folch extraction method (61) and TG levels were analyzed by the Triglyceride assay kit (Pointe Scientific), according to the manufacturer’s instructions.

### TG secretion

Mice were fasted for 4 h and injected with vehicle or Triton WR 1339 (Sigma) at a dose of 500 mg/kg BW intravenously (62). Blood samples were collected at 0, 1.5, 3, and 4 h after injection. Plasma TG levels were measured by the Triglyceride assay kit (Pointe Scientific).

### RNA isolation and analysis

Total RNA was isolated using TRIzol (Invitrogen) according to the manufacturer’s instructions. RNA was reverse-transcribed using High-Capacity cDNA Reverse Transcription Kit (Applied Biosystems) according to the manufacturer’s instructions, and the resultant cDNA was amplified and quantified using SsoAdvanced Universal SYBR Green Supermix (Bio-Rad) on the CFX 384 Real-Time System (Bio-Rad). Values were normalized to the expression levels of housekeeping genes, hypoxanthine phosphoribosyltransferase 1 (*Hprt*), ribosomal protein L32 (*Rpl32*), or small nuclear RNA U6-1 (*Rnu6-1*). The relative gene expression levels were calculated by using the ΔΔ-Ct method.

### Protein isolation and Western blotting

Total protein lysates from mouse livers were prepared in a radioimmunoprecipitation assay buffer, fractionated on 8 to 12% SDS-PAGE, and transferred to a PVDF membrane. The blot was incubated with an indicated primary antibody followed by incubation with a secondary antibody conjugated to horseradish peroxidase. Reactivity was detected with an enhanced chemiluminescence kit (Pierce). The antibodies used in this study are as follows: HNF1B and MTTP from BD Biosciences; APOO from ThermoFisher Scientific; PPARα, GAPDH, and TUBULIN from Santa Cruz Biotechnology.

### Transmission electron microscopy

Liver tissues were processed for TEM, as described previously (63, 64) with minor modifications. Briefly, 2 × 2 mm cubes of tissues were fixed at 4 °C overnight in EM grade fixative (2.5% glutaraldehyde in 0.1 M Na cacodylate buffer containing 3 mM MgCl_2_, pH 7.4) followed by three washes in buffer containing 0.1 M Na cacodylate and 3 mM MgCl_2_, pH 7.4. Tissues were postfixed in fixative (1% osmium tetroxide in 0.1 M Na cacodylate buffer, 0.8% K_3_[Fe(CN)_6_], and 3 mM MgCl_2_, pH 7.4) for 1 h at room temperature, followed by dehydration and propylene oxide exposure. Tissues were then embedded in glauert medium resin, polymerized at 60 °C for 48 h, sectioned, and collected on grids. Grids were stained with 1.5% ethanolic uranyl acetate and 2.5% Sato’s lead citrate. Images were obtained using an FEI Tecnai T12 transmission electron microscope equipped with an AMT 2K XR40 CCD camera at an accelerating voltage of 80 KV. TEM was performed at the Bioscience Electron Microscopy Laboratory at the University of Connecticut.

### Primary hepatocyte isolation

Primary mouse hepatocytes were isolated as described previously (15). Briefly, hepatocytes were isolated by a 2-step collagenase perfusion method from WT (WT-EtOH)- and KO (KO-EtOH)-mice–fed LDC supplemented with ethanol (5% vol/vol) for 1 month. Viable hepatocytes were purified by percoll (GE Healthcare) gradient centrifugation.

### Oxygen consumption rate analysis

The real-time oxygen consumption rate was analyzed using the Seahorse XF Mito Stress Test in Seahorse XF24 analyzer (Agilent Technologies). WT-EtOH and KO-EtOH hepatocytes were plated at 1.5 × 10^4^/well in collagen-coated XF24 plates. After a three-hour attachment, the media were replaced and hepatocytes were cultured overnight in fresh media containing 100 mM ethanol. On the next day, hepatocytes were washed with XF media and incubated in a non-CO_2_ incubator at 37 °C for 1 h. Analyses were carried out both at basal conditions and after the sequential injections of inhibitors: 1 μM oligomycin, 1 μM carbonyl cyanide-4 (trifluoromethoxy) phenylhydrazone, and a mixture of 1 μM antimycin A & 1 μM of rotenone. Data were normalized to DNA concentrations.

### Metabolomics and lipidomics analyses

Fifty micrograms of snap frozen liver samples from WT- (n = 8) and KO- (n = 7) mice–fed EB were processed for untargeted screening for metabolome. Analyses of metabolomics and lipidomics were performed at the West Coast Metabolomics Center, University of California, Davis, as described previously (15). Bioinformatic analysis of the data was performed using MetaboAnalyst 3.0 software.

### Immunofluorescence

Immunofluorescence was performed as described previously (65). Briefly, paraffin-embedded liver slides were dewaxed, antigen-retrieved, and permeabilized followed by blocking in 5% bovine serum albumin. Slides were incubated with a primary antibody (LC3A/B, Cell Signaling Technology) overnight at room temperature followed by washes and secondary antibody (Alexa fluor 488, ThermoFisher Scientific) incubation. Slides were washed and mounted with DAPI mounting medium (Invitrogen). Images were obtained by Nikon A1R Confocal microscope using the 40× oil immersion objective.

### Statistical analysis

Data are represented as the mean ± the SEM. Statistical differences were assessed by using a two-tailed unequal variance of Student *t* test between two groups and one-way ANOVA among multiple groups. A *p* value less than 0.05 (*p* < 0.05) was considered statistically significant.

## Data availability

All data supporting the findings of this study are contained within the article.

## Supporting information

This article contains supporting information (16).

## Conflict of interest

The authors declare that they have no conflicts of interest with the contents of this article.

## References

[1] O'Shea R.S., Dasarathy S., McCullough A.J. (2010). Alcoholic liver disease. Hepatology.

[2] Gao B., Bataller R. (2011). Alcoholic liver disease: Pathogenesis and new therapeutic targets. Gastroenterology.

[3] Clugston R.D., Yuen J.J., Hu Y., Abumrad N.A., Berk P.D., Goldberg I.J., Blaner W.S., Huang L.S. (2014). CD36-deficient mice are resistant to alcohol- and high-carbohydrate-induced hepatic steatosis. J. Lipid Res..

[4] You M., Fischer M., Deeg M.A., Crabb D.W. (2002). Ethanol induces fatty acid synthesis pathways by activation of sterol regulatory element-binding protein (SREBP). J. Biol. Chem..

[5] Yu J.H., Song S.J., Kim A., Choi Y., Seok J.W., Kim H.J., Lee Y.J., Lee K.S., Kim J.W. (2016). Suppression of PPARgamma-mediated monoacylglycerol O-acyltransferase 1 expression ameliorates alcoholic hepatic steatosis. Sci. Rep..

[6] Xu M.J., Cai Y., Wang H., Altamirano J., Chang B., Bertola A., Odena G., Lu J., Tanaka N., Matsusue K., Matsubara T., Mukhopadhyay P., Kimura S., Pacher P., Gonzalez F.J. (2015). Fat-specific protein 27/CIDEC promotes development of alcoholic steatohepatitis in mice and humans. Gastroenterology.

[7] Carr R.M., Peralta G., Yin X., Ahima R.S. (2014). Absence of perilipin 2 prevents hepatic steatosis, glucose intolerance and ceramide accumulation in alcohol-fed mice. PLoS one.

[8] Fischer M., You M., Matsumoto M., Crabb D.W. (2003). Peroxisome proliferator-activated receptor alpha (PPARalpha) agonist treatment reverses PPARalpha dysfunction and abnormalities in hepatic lipid metabolism in ethanol-fed mice. J. Biol. Chem..

[9] Venkatesan S., Ward R.J., Peters T.J. (1988). Effect of chronic ethanol feeding on the hepatic secretion of very-low-density lipoproteins. Biochim. Biophys. Acta.

[10] Sugimoto T., Yamashita S., Ishigami M., Sakai N., Hirano K., Tahara M., Matsumoto K., Nakamura T., Matsuzawa Y. (2002). Decreased microsomal triglyceride transfer protein activity contributes to initiation of alcoholic liver steatosis in rats. J. Hepatol..

[11] Feng X., Wang Z., Fillmore R., Xi Y. (2014). MiR-200, a new star miRNA in human cancer. Cancer Lett..

[12] Hasuwa H., Ueda J., Ikawa M., Okabe M. (2013). miR-200b and miR-429 function in mouse ovulation and are essential for female fertility. Science.

[13] Belgardt B.F., Ahmed K., Spranger M., Latreille M., Denzler R., Kondratiuk N., von Meyenn F., Villena F.N., Herrmanns K., Bosco D., Kerr-Conte J., Pattou F., Rülicke T., Stoffel M. (2015). The microRNA-200 family regulates pancreatic beta cell survival in type 2 diabetes. Nat. Med..

[14] Cao H., Jheon A., Li X., Sun Z., Wang J., Florez S., Zhang Z., McManus M.T., Klein O.D., Amendt B.A. (2013). The Pitx2:miR-200c/141:noggin pathway regulates Bmp signaling and ameloblast differentiation. Development.

[15] Tran M., Lee S.M., Shin D.J., Wang L. (2017). Loss of miR-141/200c ameliorates hepatic steatosis and inflammation by reprogramming multiple signaling pathways in NASH. JCI insight.

[16] Bertola A., Mathews S., Ki S.H., Wang H., Gao B. (2013). Mouse model of chronic and binge ethanol feeding (the NIAAA model). Nat. Protoc..

[17] Ki S.H., Park O., Zheng M., Morales-Ibanez O., Kolls J.K., Bataller R., Gao B. (2010). Interleukin-22 treatment ameliorates alcoholic liver injury in a murine model of chronic-binge ethanol feeding: Role of signal transducer and activator of transcription 3. Hepatology.

[18] Hussain M.M., Nijstad N., Franceschini L. (2011). Regulation of microsomal triglyceride transfer protein. Clin. Lipidol..

[19] Lewis B.P., Shih I.H., Jones-Rhoades M.W., Bartel D.P., Burge C.B. (2003). Prediction of mammalian microRNA targets. Cell.

[20] Yao Z.M., Vance D.E. (1988). The active synthesis of phosphatidylcholine is required for very low density lipoprotein secretion from rat hepatocytes. J. Biol. Chem..

[21] Vance D.E. (1990). Boehringer mannheim award lecture. Phosphatidylcholine metabolism: Masochistic enzymology, metabolic regulation, and lipoprotein assembly. Biochem. Cel. Biol..

[22] Koob S., Reichert A.S. (2014). Novel intracellular functions of apolipoproteins: The ApoO protein family as constituents of the mitofilin/MINOS complex determines cristae morphology in mitochondria. Biol. Chem..

[23] Hoek J.B., Cahill A., Pastorino J.G. (2002). Alcohol and mitochondria: A dysfunctional relationship. Gastroenterology.

[24] Lee Y.K., Lee J.A. (2016). Role of the mammalian ATG8/LC3 family in autophagy: Differential and compensatory roles in the spatiotemporal regulation of autophagy. BMB Rep..

[25] Grygiel-Gorniak B. (2014). Peroxisome proliferator-activated receptors and their ligands: Nutritional and clinical implications--a review. Nutr. J..

[26] Kersten S., Seydoux J., Peters J.M., Gonzalez F.J., Desvergne B., Wahli W. (1999). Peroxisome proliferator-activated receptor alpha mediates the adaptive response to fasting. J. Clin. Invest..

[27] Lin M.C., Li J.J., Wang E.J., Princler G.L., Kauffman F.C., Kung H.F. (1997). Ethanol down-regulates the transcription of microsomal triglyceride transfer protein gene. FASEB J..

[28] Horikawa Y., Iwasaki N., Hara M., Furuta H., Hinokio Y., Cockburn B.N., Lindner T., Yamagata K., Ogata M., Tomonaga O., Kuroki H., Kasahara T., Iwamoto Y., Bell G.I. (1997). Mutation in hepatocyte nuclear factor-1 beta gene (TCF2) associated with MODY. Nat. Genet..

[29] Han X., Luo Y., Ren Q., Zhang X., Wang F., Sun X., Zhou X., Ji L. (2010). Implication of genetic variants near SLC30A8, HHEX, CDKAL1, CDKN2A/B, IGF2BP2, FTO, TCF2, KCNQ1, and WFS1 in type 2 diabetes in a Chinese population. BMC Med. Genet..

[30] Kornfeld J.W., Baitzel C., Konner A.C., Nicholls H.T., Vogt M.C., Herrmanns K., Scheja L., Haumaitre C., Wolf A.M., Knippschild U., Seibler J., Cereghini S., Heeren J., Stoffel M., Brüning J.C. (2013). Obesity-induced overexpression of miR-802 impairs glucose metabolism through silencing of Hnf1b. Nature.

[31] Long Z., Cao M., Su S., Wu G., Meng F., Wu H., Liu J., Yu W., Atabai K., Wang X. (2017). Inhibition of hepatocyte nuclear factor 1b induces hepatic steatosis through DPP4/NOX1-mediated regulation of superoxide. Free Radic. Biol. Med..

[32] Hayhurst G.P., Lee Y.H., Lambert G., Ward J.M., Gonzalez F.J. (2001). Hepatocyte nuclear factor 4alpha (nuclear receptor 2A1) is essential for maintenance of hepatic gene expression and lipid homeostasis. Mol. Cell. Biol..

[33] Thumser A.E., Moore J.B., Plant N.J. (2014). Fatty acid binding proteins: Tissue-specific functions in health and disease. Curr. Opin. Clin. Nutr. Metab. Care.

[34] Smathers R.L., Galligan J.J., Shearn C.T., Fritz K.S., Mercer K., Ronis M., Orlicky D.J., Davidson N.O., Petersen D.R. (2013). Susceptibility of L-FABP-/- mice to oxidative stress in early-stage alcoholic liver. J. Lipid Res..

[35] Gaemers I.C., Stallen J.M., Kunne C., Wallner C., van Werven J., Nederveen A., Lamers W.H. (2011). Lipotoxicity and steatohepatitis in an overfed mouse model for non-alcoholic fatty liver disease. Biochim. Biophys. Acta.

[36] Charlton M., Viker K., Krishnan A., Sanderson S., Veldt B., Kaalsbeek A.J., Kendrick M., Thompson G., Que F., Swain J., Sarr M. (2009). Differential expression of lumican and fatty acid binding protein-1: New insights into the histologic spectrum of nonalcoholic fatty liver disease. Hepatology.

[37] Attal N., Sullivan M.T., Girardi C.A., Thompson K.J., McKillop I.H. (2021). Fatty acid binding protein-4 promotes alcohol-dependent hepatosteatosis and hepatocellular carcinoma progression. Transl. Oncol..

[38] Mavrelis P.G., Ammon H.V., Gleysteen J.J., Komorowski R.A., Charaf U.K. (1983). Hepatic free fatty acids in alcoholic liver disease and morbid obesity. Hepatology.

[39] Ontko J.A. (1973). Effects of ethanol on the metabolism of free fatty acids in isolated liver cells. J. Lipid Res..

[40] Sampath H., Ntambi J.M. (2005). The fate and intermediary metabolism of stearic acid. Lipids.

[41] Jandacek R.J. (2017). Linoleic acid: A nutritional quandary. Healthcare (Basel).

[42] Feng Y.Y., Xu X.Q., Ji C.B., Shi C.M., Guo X.R., Fu J.F. (2014). Aberrant hepatic microRNA expression in nonalcoholic fatty liver disease. Cell Physiol. Biochem..

[43] Kennell J.A., Gerin I., MacDougald O.A., Cadigan K.M. (2008). The microRNA miR-8 is a conserved negative regulator of Wnt signaling. Proc. Natl. Acad. Sci. U. S. A..

[44] Zhang G., Zhang W., Li B., Stringer-Reasor E., Chu C., Sun L., Bae S., Chen D., Wei S., Jiao K., Yang W.H., Cui R., Liu R., Wang L. (2017). MicroRNA-200c and microRNA- 141 are regulated by a FOXP3-KAT2B axis and associated with tumor metastasis in breast cancer. Breast Cancer Res..

[45] Burk U., Schubert J., Wellner U., Schmalhofer O., Vincan E., Spaderna S., Brabletz T. (2008). A reciprocal repression between ZEB1 and members of the miR-200 family promotes EMT and invasion in cancer cells. EMBO Rep..

[46] Koob S., Barrera M., Anand R., Reichert A.S. (2015). The non-glycosylated isoform of MIC26 is a constituent of the mammalian MICOS complex and promotes formation of crista junctions. Biochim. Biophys. Acta.

[47] Lamant M., Smih F., Harmancey R., Philip-Couderc P., Pathak A., Roncalli J., Galinier M., Collet X., Massabuau P., Senard J.M., Rouet P. (2006). ApoO, a novel apolipoprotein, is an original glycoprotein up-regulated by diabetes in human heart. J. Biol. Chem..

[48] Nijstad N., de Boer J.F., Lagor W.R., Toelle M., Usher D., Annema W., der Giet M., Rader D.J., Tietge U.J. (2011). Overexpression of apolipoprotein O does not impact on plasma HDL levels or functionality in human apolipoprotein A-I transgenic mice. Biochim. Biophys. Acta.

[49] Tian F., Wu C.L., Yu B.L., Liu L., Hu J.R. (2017). Apolipoprotein O expression in mouse liver enhances hepatic lipid accumulation by impairing mitochondrial function. Biochem. Biophys. Res. Commun..

[50] Jacobs R.L., Devlin C., Tabas I., Vance D.E. (2004). Targeted deletion of hepatic CTP:phosphocholine cytidylyltransferase alpha in mice decreases plasma high density and very low density lipoproteins. J. Biol. Chem..

[51] Lieber C.S., Robins S.J., Leo M.A. (1994). Hepatic phosphatidylethanolamine methyltransferase activity is decreased by ethanol and increased by phosphatidylcholine. Alcohol. Clin. Exp. Res..

[52] Noga A.A., Vance D.E. (2003). A gender-specific role for phosphatidylethanolamine N-methyltransferase-derived phosphatidylcholine in the regulation of plasma high density and very low density lipoproteins in mice. J. Biol. Chem..

[53] Schapiro R.H., Scheig R.L., Drummey G.D., Mendelson J.H., Isselbacher K.J. (1965). Effect of prolonged ethanol ingestion on the transport and metabolism of lipids in man. New Engl. J. Med..

[54] Morland J. (1974). Effect of chronic ethanol treatment on tryptophan oxygenase, tyrosine aminotransferase and general protein metabolism in the intact and perfused rat liver. Biochem. Pharmacol..

[55] Losowsky M.S., Jones D.P., Davidson C.S., Lieber C.S. (1963). Studies of alcoholic hyperlipemia and its mechanism. Am. J. Med..

[56] Baraona E., Lieber C.S. (1970). Efcts of chronic ethanol feeding on serum lipoprotein metabolism in the rat. J. Clin. Invest..

[57] Schapiro R.H., Drummey G.D., Shimizu Y., Isselbacher K.J. (1964). Studies on the pathogenesis of the ethanol-induced fatty liver. Ii. Effect of ethanol on palmitate-1-C-14 metabolism by the isolated perfused rat liver. J. Clin. Invest..

[58] Gordon E.R. (1972). Effect of an intoxicating dose of ethanol on lipid metabolism in an isolated perfused rat liver. Biochem. Pharmacol..

[59] Baraona E., Lieber C.S. (1979). Effects of ethanol on lipid metabolism. J. Lipid Res..

[60] Ohkawa H., Ohishi N., Yagi K. (1979). Assay for lipid peroxides in animal tissues by thiobarbituric acid reaction. Anal. Biochem..

[61] Folch J., Lees M., Sloane Stanley G.H. (1957). A simple method for the isolation and purification of total lipides from animal tissues. J. Biol. Chem..

[62] Borensztajn J., Rone M.S., Kotlar T.J. (1976). The inhibition *in vivo* of lipoprotein lipase (clearing-factor lipase) activity by triton WR-1339. Biochem. J..

[63] Das S., Hajnoczky N., Antony A.N., Csordas G., Gaspers L.D., Clemens D.L., Hoek J.B., Hajnoczky G. (2012). Mitochondrial morphology and dynamics in hepatocytes from normal and ethanol-fed rats. Pflugers Arch..

[64] Chen L., Ilham S.J., Guo T., Emadi S., Feng B. (2017). *In vitro* multichannel single-unit recordings of action potentials from mouse sciatic nerve. Biomed. Phys. Eng. Exp..

[65] Zaqout S., Becker L.L., Kaindl A.M. (2020). Immunofluorescence staining of paraffin sections step by step. Front. Neuroanat..

