# Supplementary material for: MicroRNA-200c coordinates HNF1 homeobox B and apolipoprotein O functions to modulate lipid homeostasis in alcoholic fatty liver disease
Source: J Biol Chem. 2022 Apr 20;298(6):101966. doi: 10.1016/j.jbc.2022.101966 (PMC9127369; doi:10.1016/j.jbc.2022.101966)
Supplement: Supplemental Figures S1–S6 and Table S1 [file mmc1.docx]

**Supporting Information**

**MicroRNA-200c coordinates HNF1 homeobox B and Apolipoprotein O functions to modulate lipid homeostasis in alcoholic fatty liver disease**

Md Golam Mostofa, Melanie Tran, Shaynian Gilling, Grace Lee, Ondine Fraher, Lei Jin, Hyunju Kang, Young-Ki Park, Ji-Young Lee, Li Wang, Dong-Ju Shin


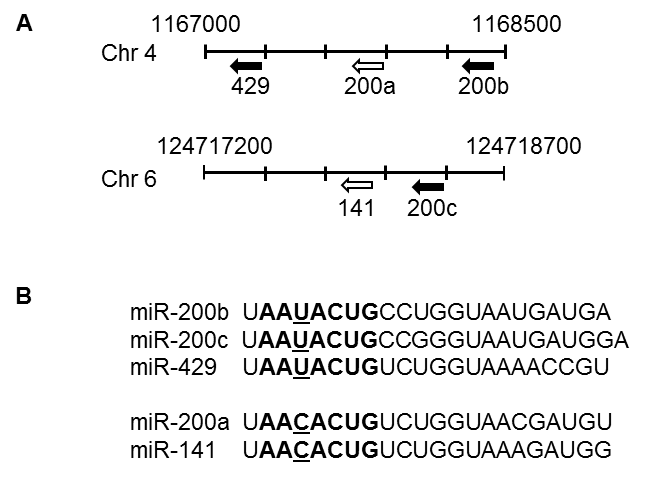


**Figure S1. (A) Genomic locations of the miR-200 family members in mice**. miR-429, -200c, and 200b are clustered on chromosome 4, whereas miR-141 and -200c are located on chromosome 6. **(B) Seed sequences of the miR-200c family members**. miR-200b, -200c, and 429 share the same seed sequence, while miR-200a and -141 share the same seed sequence. Seed sequences are shown in bold. Underlined characters represent the nucleotides that are different between the two groups.


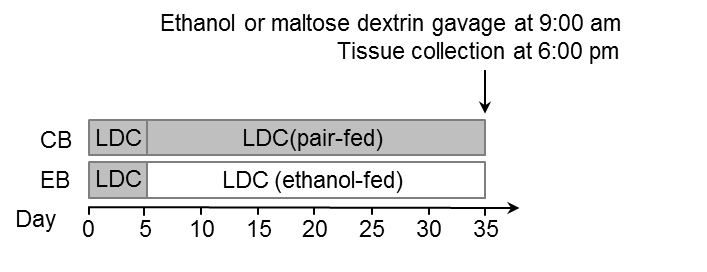


**Figure S2**. **A schematic view of mouse feeding procedure.** Both groups of mice were fed an LDC for 5 days followed by either the LDC or the LDC containing ethanol for 30 days. On the thirty-first day, mice were treated with maltose dextrin or ethanol by oral gavage of single binge at 9:00 am and sacrificed at 6:00 pm without removing feeding tubes from the cages (16). LDC, Lieber-DeCarli liquid diet; CB, LDC without ethanol supplementation plus maltose dextrin binge; EB, LDC supplemented with ethanol plus ethanol binge.


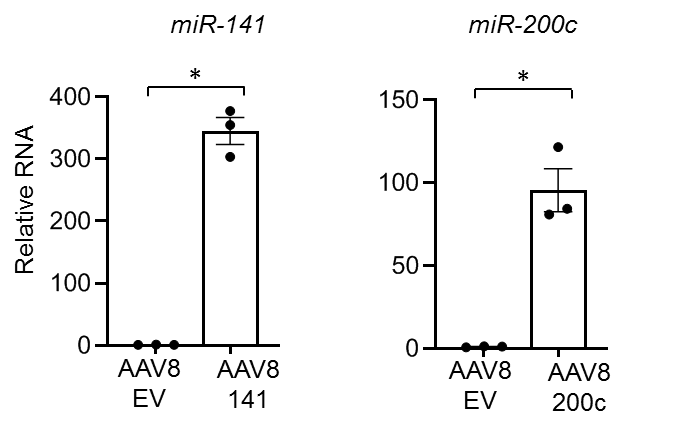


**Figure S3**. **Verification of miR-141 and -200c overexpression in mice transduced with AAV8-miR-141 or AAV8-miR-200c.** RNA expression levels of miR-141 or -200c were measured by qPCR with pooled RNA from mouse livers and normalized to *Rnu6-1*. Data are presented as mean ± SEM. Statistical significance was assessed by Student’s *t* test; *, P < 0.05. EV, empty vector

**
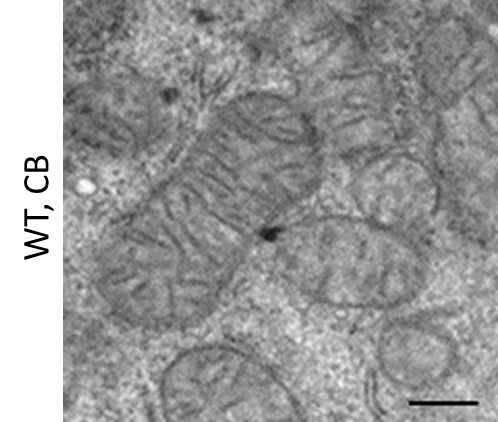

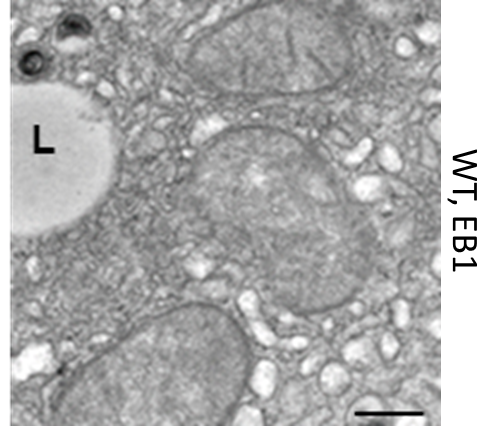
**

**
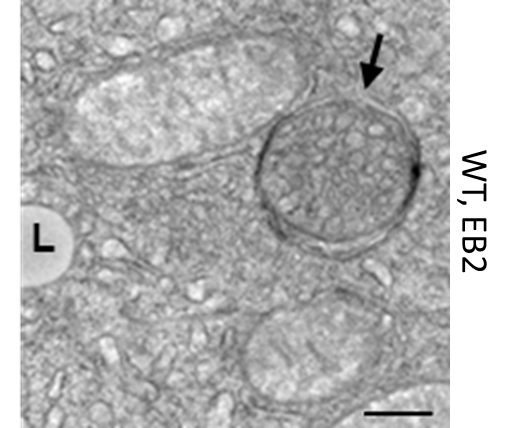

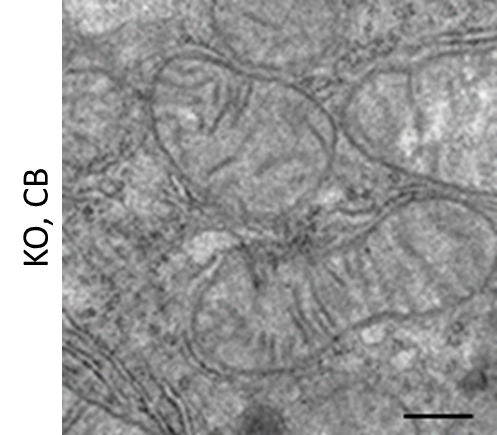
**

**
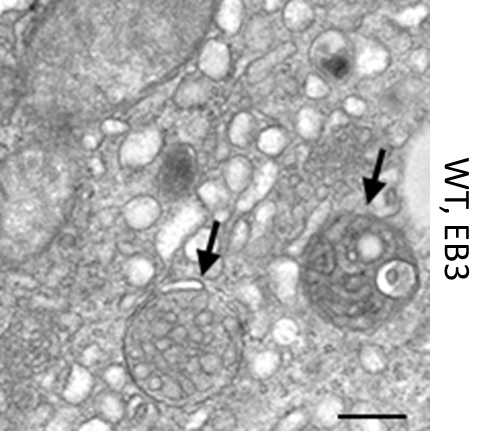

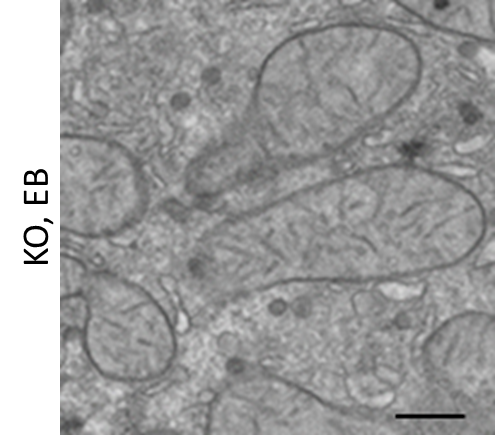
**

**Figure S4. Enlarged images of Fig. 7A.** An additional image of WT, EB3 is shown. L, lipid droplet. Arrows indicate autophagosomes. Scale bar, 500 nm.

**
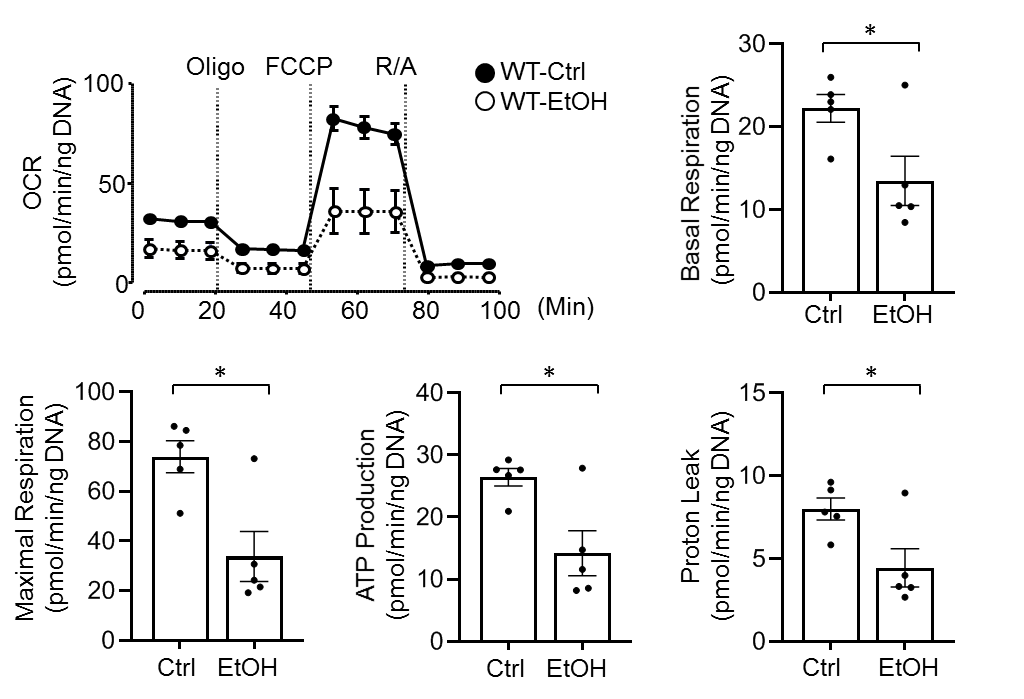
**

**Figure S5. Impaired mitochondrial function by chronic ethanol exposure.** OCR of primary hepatocytes prepared from WT fed the LDC supplemented without (WT-Ctrl) or with ethanol (WT-EtOH) for one month was analyzed by Seahorse FX24 analyzer under basal conditions or in response to indicated inhibitors. Data are presented as mean ± SEM. Statistical significance was assessed by Student’s *t* test; *, *P* < 0.05. OCR, oxygen consumption rate; Oligo, oligomycin; FCCP, Carbonyl cyanide-4 (trifluoromethoxy) phenylhydrazone; R/A, rotenone and antimycin A.


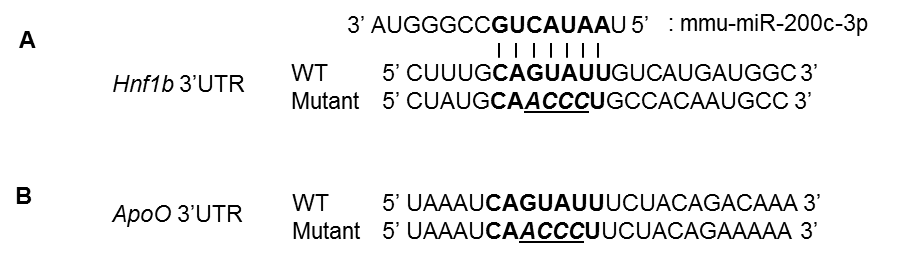


**Figure S6. Generation of WT and mutant of *Hnf1b*-3’UTR and *ApoO*-3’UTR luciferase reporters** Mutant sequences introduced to the putative miR-200c seed sequence are shown in underlined italic characters for *Hnf1b* 3’ UTR (A) and *ApoO* 3’ UTR (B). The seed sequence of miR-200c is shown in bold.


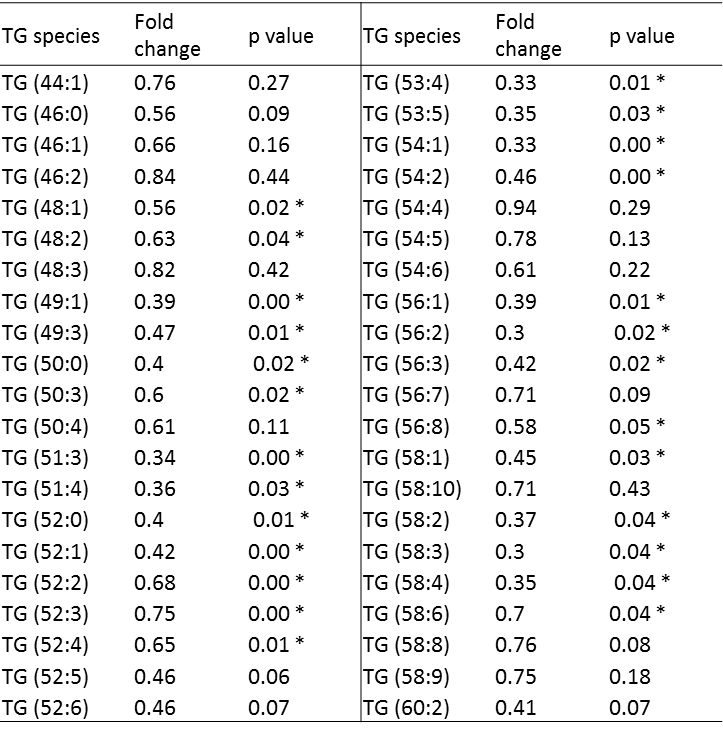


**Table S1**. **Reduced hepatic TG species in KO-EB compared to WT-EB.** Different TG species were examined in livers of WT-EB and KO-EB by metabolomics and lipidomics analyses (n=7-8). Statistical significance was assessed by Student’s *t* test; *, P < 0.05

**References**

16. A. Bertola, S. Mathews, S.H. Ki, H. Wang and B. Gao, Mouse model of chronic and binge ethanol feeding (the NIAAA model), Nat. Protoc., 8, 2013, 627–637.
